# Possible modulation of the amplitude and frequency of resting parkinsonian tremor by touching the trapezius muscle

**DOI:** 10.1002/ccr3.2893

**Published:** 2020-04-23

**Authors:** Takafumi Hasegawa, Shun Ishiyama, Takaaki Nakamura

**Affiliations:** ^1^ Division of Neurology Department of Neuroscience & Sensory Organs Tohoku University Graduate School of Medicine Sendai Japan

**Keywords:** forelimb, Parkinson's disease, propriospinal neuron, resting tremor, trapezius muscle

## Abstract

The pathophysiological mechanism of resting tremor in Parkinson's disease remains obscure. Spinal/peripheral mechanisms may modulate oscillatory activity from central origin, thereby changing amplitude and frequency of tremor in Parkinson's disease.

## Graphical abstract

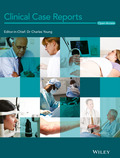

Tremor is one of the most recognizable clinical signs of Parkinson's disease. Although the central oscillation from the basal ganglia and the cerebello‐thalamo‐cortical circuit is the most accepted theory in tremor generation, spinal/peripheral mechanisms would have some roles in the modulation and manifestation of parkinsonian tremor.

A 62‐year‐old woman with Parkinson's disease (PD) happened to realize that resting tremor in her left arm was inducible by ipsilateral shoulder massage (Video S1). Her limb shaking could be augmented by other person as well (Video S2). The trigger point in this case was the trapezius muscle, which receives proprioceptive fibers from the C3‐C4 cervical nerves. This seems interesting because propriospinal neurons in the middle spinal cord mediate cortical oscillatory commands and project to forelimb motor neurons.^1^ Although central oscillations are the most accepted theory in resting tremor generation, spinal/peripheral mechanisms would have clinicopathological impact on Parkinsonian tremor.^2^

## CONFLICT OF INTEREST

None declared.

## AUTHOR CONTRIBUTIONS

TH: is the main investigator of this study and contributed to study design. SI and TN: involved in the acquisition of data.

## Supporting information

Video S1

Video S2
